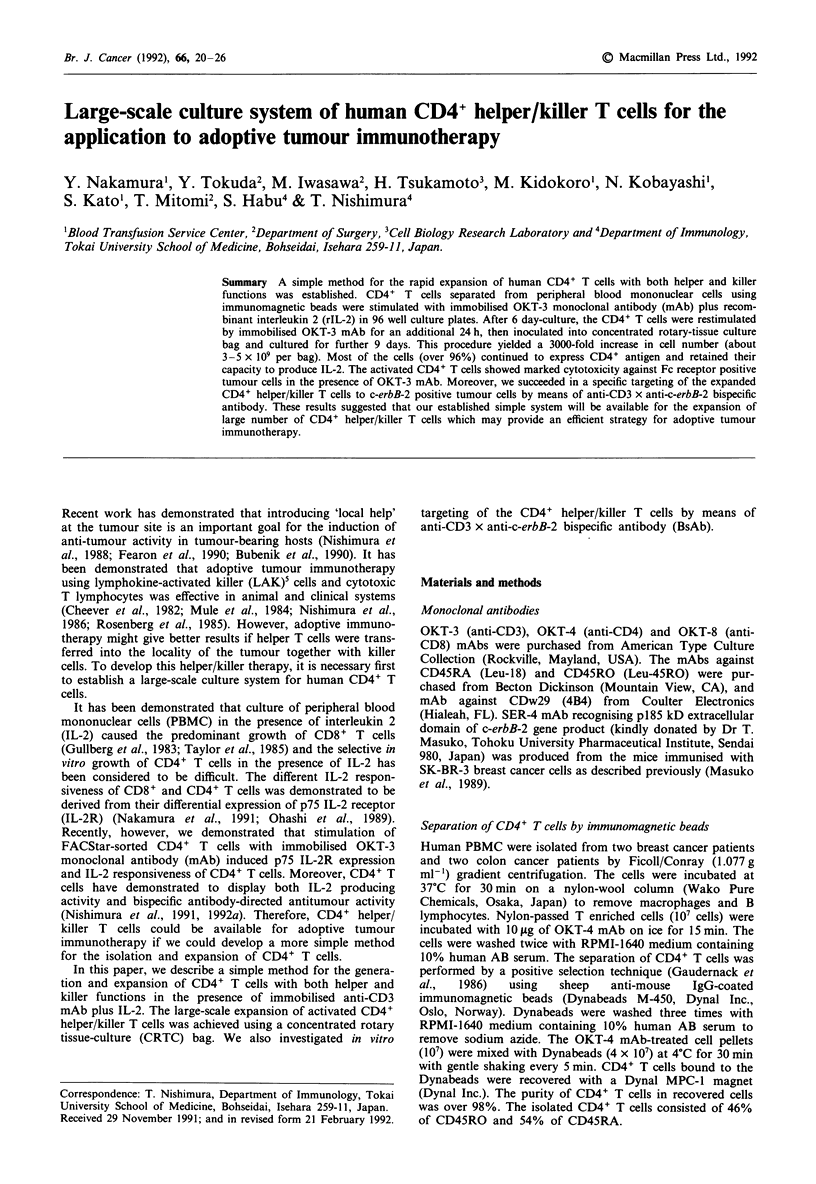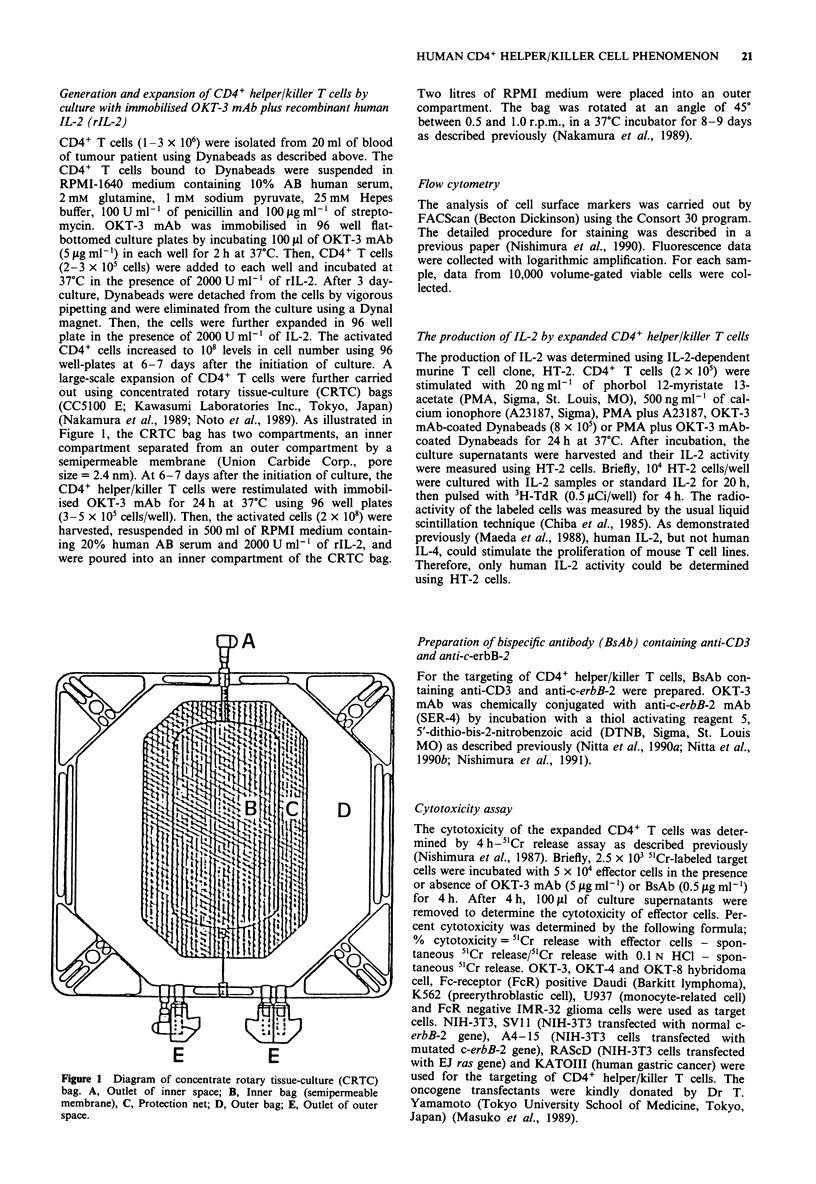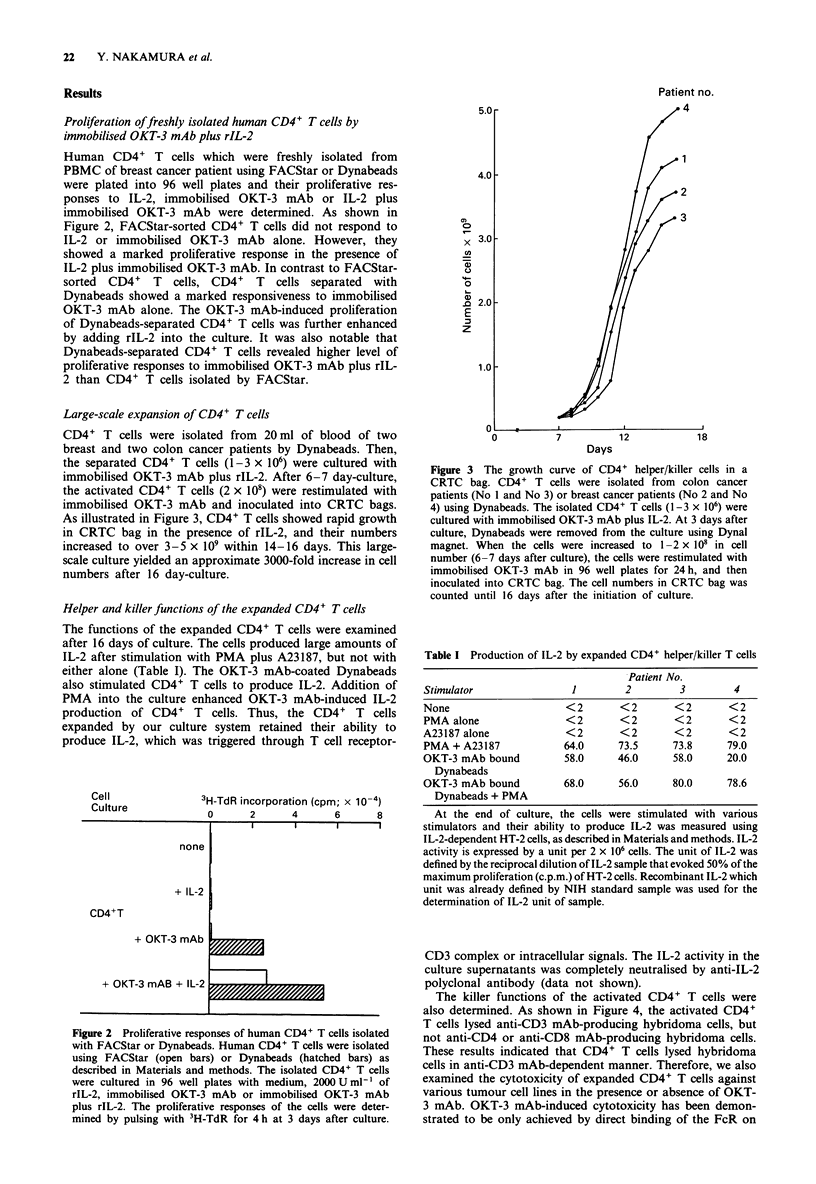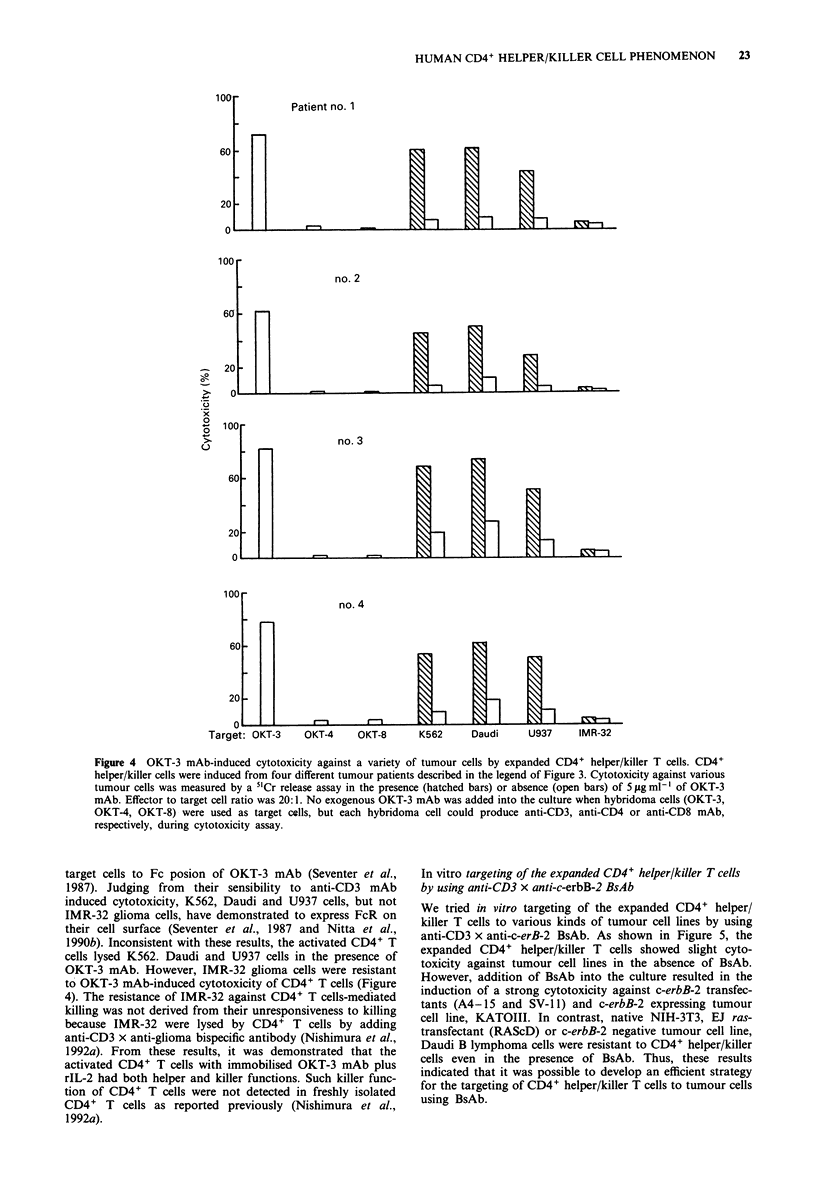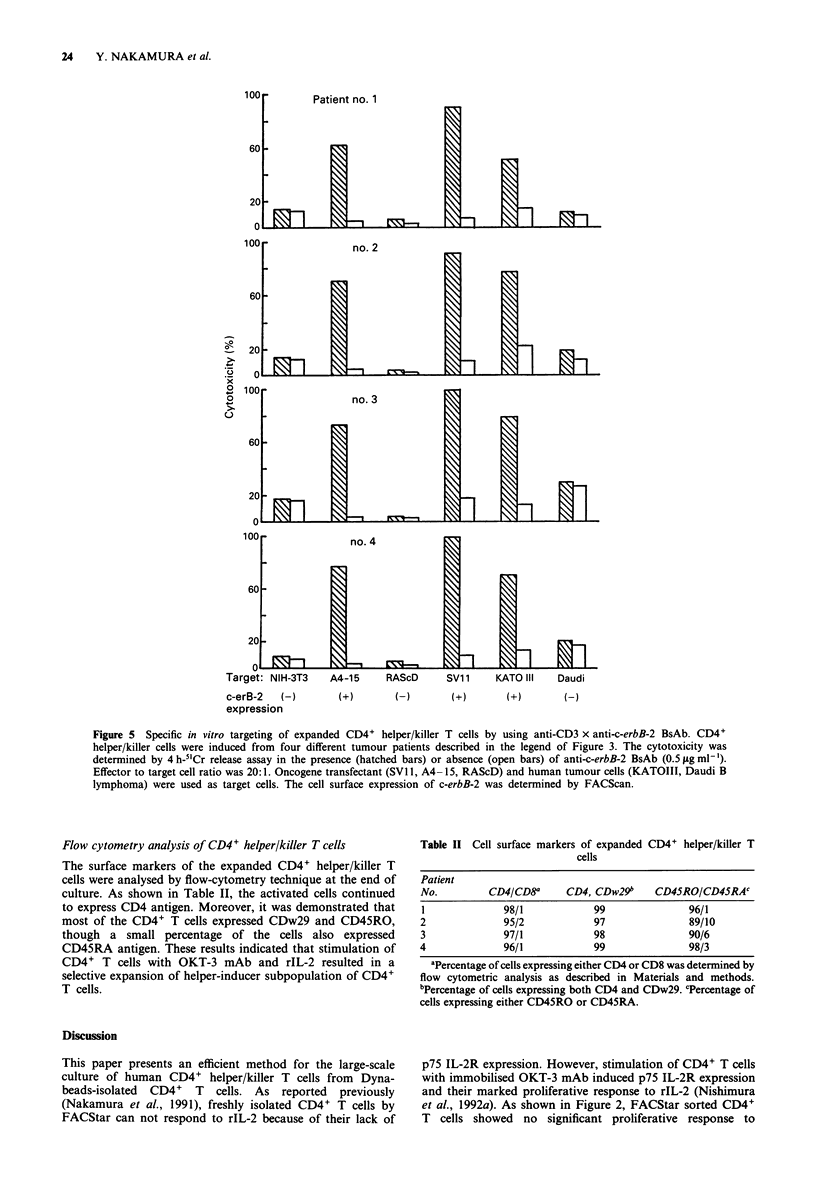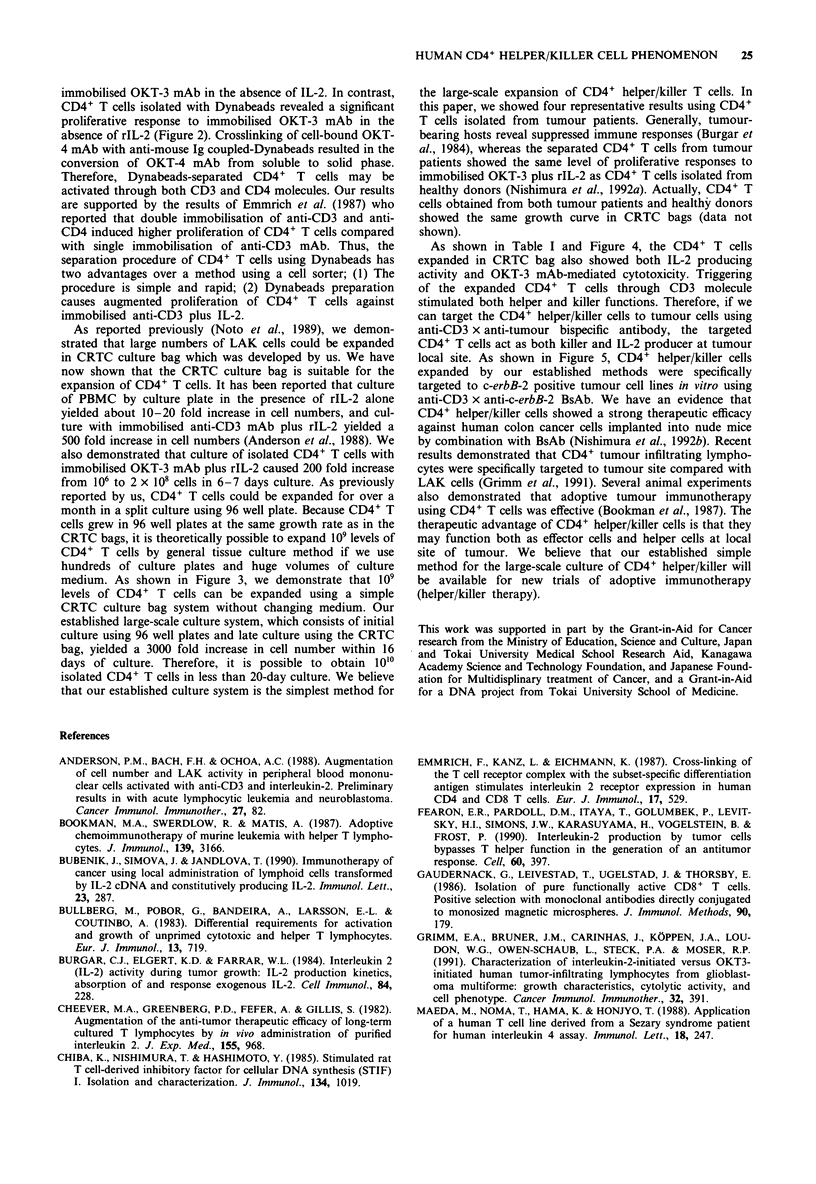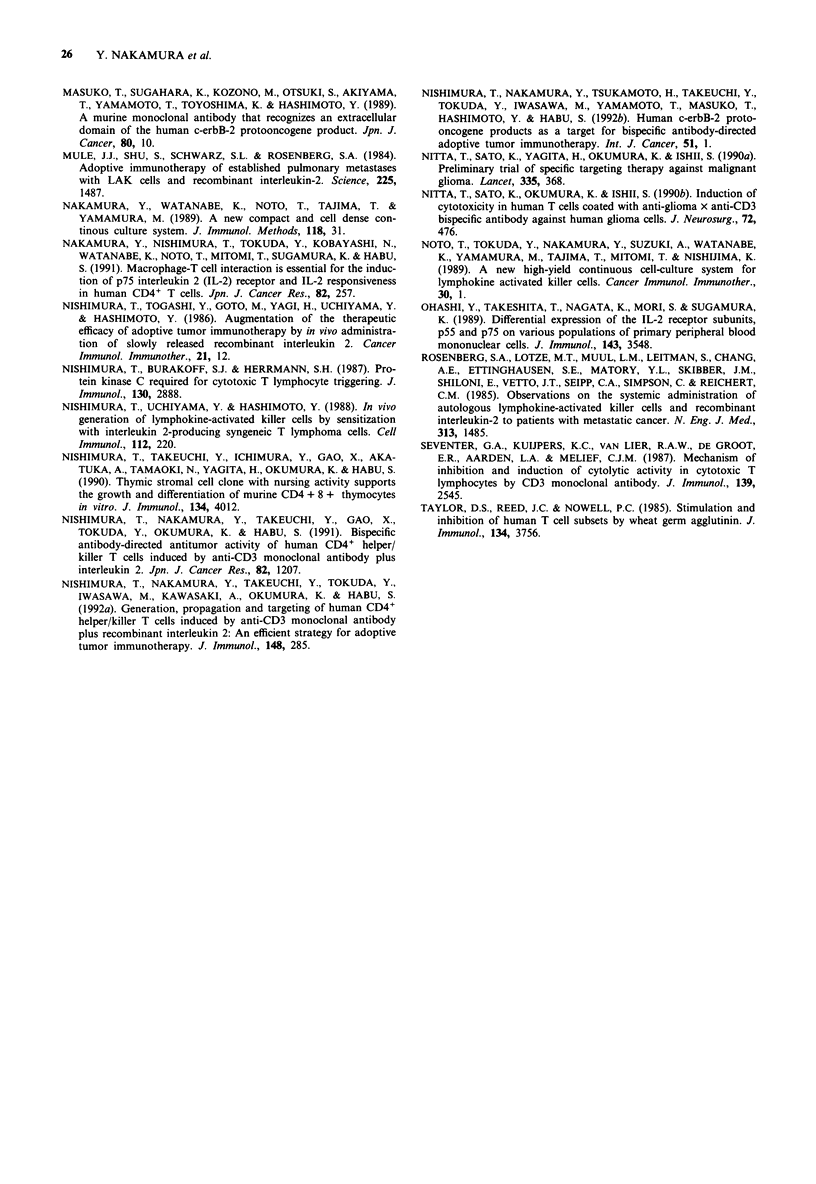# Large-scale culture system of human CD4+ helper/killer T cells for the application to adoptive tumour immunotherapy.

**DOI:** 10.1038/bjc.1992.210

**Published:** 1992-07

**Authors:** Y. Nakamura, Y. Tokuda, M. Iwasawa, H. Tsukamoto, M. Kidokoro, N. Kobayashi, S. Kato, T. Mitomi, S. Habu, T. Nishimura

**Affiliations:** Blood Transfusion Service Center, Tokai University School of Medicine, Isehara, Japan.

## Abstract

A simple method for the rapid expansion of human CD4+ T cells with both helper and killer functions was established. CD4+ T cells separated from peripheral blood mononuclear cells using immunomagnetic beads were stimulated with immobilised OKT-3 monoclonal antibody (mAb) plus recombinant interleukin 2 (rIL-2) in 96 well culture plates. After 6 day-culture, the CD4+ T cells were restimulated by immobilised OKT-3 mAb for an additional 24 h, then inoculated into concentrated rotary-tissue culture bag and cultured for further 9 days. This procedure yielded a 3000-fold increase in cell number (about 3-5 x 10(9) per bag). Most of the cells (over 96%) continued to express CD4+ antigen and retained their capacity to produce IL-2. The activated CD4+ T cells showed marked cytotoxicity against Fc receptor positive tumour cells in the presence of OKT-3 mAb. Moreover, we succeeded in a specific targeting of the expanded CD4+ helper/killer T cells to c-erb B-2 positive tumour cells by means of anti-CD3 x anti-c-erb B-2 bispecific antibody. These results suggested that our established simple system will be available for the expansion of large number of CD4+ helper/killer T cells which may provide an efficient strategy for adoptive tumour immunotherapy.


Br. J. Cancer (1992), 66, 20–26 © Macmillan Press Ltd., 1992

### Large-scale culture system of human CD4$^+$ helper/killer T cells for the application to adoptive tumour immunotherapy

Y. Nakamura[1], Y. Tokuda[2], M. Iwasawa[2], H. Tsukamoto[3], M. Kidokoro[1], N. Kobayashi[1], S. Kato[1], T. Mitomi[2], S. Habu[4] & T. Nishimura[4]

[1]*Blood Transfusion Service Center,* [2]*Department of Surgery,* [3]*Cell Biology Research Laboratory and* [4]*Department of Immunology, Tokai University School of Medicine, Bohseidai, Isehara 259-11, Japan.*

**Summary** A simple method for the rapid expansion of human CD4$^+$ T cells with both helper and killer functions was established. CD4$^+$ T cells separated from peripheral blood mononuclear cells using immunomagnetic beads were stimulated with immobilised OKT-3 monoclonal antibody (mAb) plus recombinant interleukin 2 (rIL-2) in 96 well culture plates. After 6 day-culture, the CD4$^+$ T cells were restimulated by immobilised OKT-3 mAb for an additional 24 h, then inoculated into concentrated rotary-tissue culture bag and cultured for further 9 days. This procedure yielded a 3000-fold increase in cell number (about $3-5 \times 10^9$ per bag). Most of the cells (over 96%) continued to express CD4$^+$ antigen and retained their capacity to produce IL-2. The activated CD4$^+$ T cells showed marked cytotoxicity against Fc receptor positive tumour cells in the presence of OKT-3 mAb. Moreover, we succeeded in a specific targeting of the expanded CD4$^+$ helper/killer T cells to c-*erbB*-2 positive tumour cells by means of anti-CD3 × anti-c-*erbB*-2 bispecific antibody. These results suggested that our established simple system will be available for the expansion of large number of CD4$^+$ helper/killer T cells which may provide an efficient strategy for adoptive tumour immunotherapy.

Recent work has demonstrated that introducing 'local help' at the tumour site is an important goal for the induction of anti-tumour activity in tumour-bearing hosts (Nishimura *et al.*, 1988; Fearon *et al.*, 1990; Bubenik *et al.*, 1990). It has been demonstrated that adoptive tumour immunotherapy using lymphokine-activated killer (LAK)[5] cells and cytotoxic T lymphocytes was effective in animal and clinical systems (Cheever *et al.*, 1982; Mule *et al.*, 1984; Nishimura *et al.*, 1986; Rosenberg *et al.*, 1985). However, adoptive immunotherapy might give better results if helper T cells were transferred into the locality of the tumour together with killer cells. To develop this helper/killer therapy, it is necessary first to establish a large-scale culture system for human CD4$^+$ T cells.

It has been demonstrated that culture of peripheral blood mononuclear cells (PBMC) in the presence of interleukin 2 (IL-2) caused the predominant growth of CD8$^+$ T cells (Gullberg *et al.*, 1983; Taylor *et al.*, 1985) and the selective *in vitro* growth of CD4$^+$ T cells in the presence of IL-2 has been considered to be difficult. The different IL-2 responsiveness of CD8$^+$ and CD4$^+$ T cells was demonstrated to be derived from their differential expression of p75 IL-2 receptor (IL-2R) (Nakamura *et al.*, 1991; Ohashi *et al.*, 1989). Recently, however, we demonstrated that stimulation of FACStar-sorted CD4$^+$ T cells with immobilised OKT-3 monoclonal antibody (mAb) induced p75 IL-2R expression and IL-2 responsiveness of CD4$^+$ T cells. Moreover, CD4$^+$ T cells have demonstrated to display both IL-2 producing activity and bispecific antibody-directed antitumour activity (Nishimura *et al.*, 1991, 1992*a*). Therefore, CD4$^+$ helper/killer T cells could be available for adoptive tumour immunotherapy if we could develop a more simple method for the isolation and expansion of CD4$^+$ T cells.

In this paper, we describe a simple method for the generation and expansion of CD4$^+$ T cells with both helper and killer functions in the presence of immobilised anti-CD3 mAb plus IL-2. The large-scale expansion of activated CD4$^+$ helper/killer T cells was achieved using a concentrated rotary tissue-culture (CRTC) bag. We also investigated *in vitro* targeting of the CD4$^+$ helper/killer T cells by means of anti-CD3 × anti-c-*erbB*-2 bispecific antibody (BsAb).

Correspondence: T. Nishimura, Department of Immunology, Tokai University School of Medicine, Bohseidai, Isehara 259-11, Japan.
Received 29 November 1991; and in revised form 21 February 1992.

### Materials and methods

### *Monoclonal antibodies*

OKT-3 (anti-CD3), OKT-4 (anti-CD4) and OKT-8 (anti-CD8) mAbs were purchased from American Type Culture Collection (Rockville, Mayland, USA). The mAbs against CD45RA (Leu-18) and CD45RO (Leu-45RO) were purchased from Becton Dickinson (Mountain View, CA), and mAb against CDw29 (4B4) from Coulter Electronics (Hialeah, FL). SER-4 mAb recognising p185 kD extracellular domain of c-*erbB*-2 gene product (kindly donated by Dr T. Masuko, Tohoku University Pharmaceutical Institute, Sendai 980, Japan) was produced from the mice immunised with SK-BR-3 breast cancer cells as described previously (Masuko *et al.*, 1989).

### *Separation of CD4$^+$ T cells by immunomagnetic beads*

Human PBMC were isolated from two breast cancer patients and two colon cancer patients by Ficoll/Conray (1.077 g ml$^{-1}$) gradient centrifugation. The cells were incubated at 37°C for 30 min on a nylon-wool column (Wako Pure Chemicals, Osaka, Japan) to remove macrophages and B lymphocytes. Nylon-passed T enriched cells ($10^7$ cells) were incubated with 10 μg of OKT-4 mAb on ice for 15 min. The cells were washed twice with RPMI-1640 medium containing 10% human AB serum. The separation of CD4$^+$ T cells was performed by a positive selection technique (Gaudernack *et al.*, 1986) using sheep anti-mouse IgG-coated immunomagnetic beads (Dynabeads M-450, Dynal Inc., Oslo, Norway). Dynabeads were washed three times with RPMI-1640 medium containing 10% human AB serum to remove sodium azide. The OKT-4 mAb-treated cell pellets ($10^7$) were mixed with Dynabeads ($4 \times 10^7$) at 4°C for 30 min with gentle shaking every 5 min. CD4$^+$ T cells bound to the Dynabeads were recovered with a Dynal MPC-1 magnet (Dynal Inc.). The purity of CD4$^+$ T cells in recovered cells was over 98%. The isolated CD4$^+$ T cells consisted of 46% of CD45RO and 54% of CD45RA.

*Generation and expansion of CD4$^+$ helper/killer T cells by culture with immobilised OKT-3 mAb plus recombinant human IL-2 (rIL-2)*

CD4$^+$ T cells ($1-3 \times 10^6$) were isolated from 20 ml of blood of tumour patient using Dynabeads as described above. The CD4$^+$ T cells bound to Dynabeads were suspended in RPMI-1640 medium containing 10% AB human serum, 2 mM glutamine, 1 mM sodium pyruvate, 25 mM Hepes buffer, 100 U ml$^{-1}$ of penicillin and 100 $\mu$g ml$^{-1}$ of streptomycin. OKT-3 mAb was immobilised in 96 well flat-bottomed culture plates by incubating 100 $\mu$l of OKT-3 mAb (5 $\mu$g ml$^{-1}$) in each well for 2 h at 37°C. Then, CD4$^+$ T cells ($2-3 \times 10^5$ cells) were added to each well and incubated at 37°C in the presence of 2000 U ml$^{-1}$ of rIL-2. After 3 day-culture, Dynabeads were detached from the cells by vigorous pipetting and were eliminated from the culture using a Dynal magnet. Then, the cells were further expanded in 96 well plate in the presence of 2000 U ml$^{-1}$ of IL-2. The activated CD4$^+$ cells increased to $10^8$ levels in cell number using 96 well-plates at 6–7 days after the initiation of culture. A large-scale expansion of CD4$^+$ T cells were further carried out using concentrated rotary tissue-culture (CRTC) bags (CC5100 E; Kawasumi Laboratories Inc., Tokyo, Japan) (Nakamura *et al.*, 1989; Noto *et al.*, 1989). As illustrated in Figure 1, the CRTC bag has two compartments, an inner compartment separated from an outer compartment by a semipermeable membrane (Union Carbide Corp., pore size = 2.4 nm). At 6–7 days after the initiation of culture, the CD4$^+$ helper/killer T cells were restimulated with immobilised OKT-3 mAb for 24 h at 37°C using 96 well plates ($3-5 \times 10^5$ cells/well). Then, the activated cells ($2 \times 10^8$) were harvested, resuspended in 500 ml of RPMI medium containing 20% human AB serum and 2000 U ml$^{-1}$ of rIL-2, and were poured into an inner compartment of the CRTC bag. Two litres of RPMI medium were placed into an outer compartment. The bag was rotated at an angle of 45° between 0.5 and 1.0 r.p.m., in a 37°C incubator for 8–9 days as described previously (Nakamura *et al.*, 1989).

**Figure 1** Diagram of concentrate rotary tissue-culture (CRTC) bag. **A**, Outlet of inner space; **B**, Inner bag (semipermeable membrane), C, Protection net; **D**, Outer bag; **E**, Outlet of outer space.

*Flow cytometry*

The analysis of cell surface markers was carried out by FACScan (Becton Dickinson) using the Consort 30 program. The detailed procedure for staining was described in a previous paper (Nishimura *et al.*, 1990). Fluorescence data were collected with logarithmic amplification. For each sample, data from 10,000 volume-gated viable cells were collected.

*The production of IL-2 by expanded CD4$^+$ helper/killer T cells*

The production of IL-2 was determined using IL-2-dependent murine T cell clone, HT-2. CD4$^+$ T cells ($2 \times 10^5$) were stimulated with 20 ng ml$^{-1}$ of phorbol 12-myristate 13-acetate (PMA, Sigma, St. Louis, MO), 500 ng ml$^{-1}$ of calcium ionophore (A23187, Sigma), PMA plus A23187, OKT-3 mAb-coated Dynabeads ($8 \times 10^5$) or PMA plus OKT-3 mAb-coated Dynabeads for 24 h at 37°C. After incubation, the culture supernatants were harvested and their IL-2 activity were measured using HT-2 cells. Briefly, $10^4$ HT-2 cells/well were cultured with IL-2 samples or standard IL-2 for 20 h, then pulsed with $^3$H-TdR (0.5 $\mu$Ci/well) for 4 h. The radioactivity of the labeled cells was measured by the usual liquid scintillation technique (Chiba *et al.*, 1985). As demonstrated previously (Maeda *et al.*, 1988), human IL-2, but not human IL-4, could stimulate the proliferation of mouse T cell lines. Therefore, only human IL-2 activity could be determined using HT-2 cells.

*Preparation of bispecific antibody (BsAb) containing anti-CD3 and anti-c-*erbB-*2*

For the targeting of CD4$^+$ helper/killer T cells, BsAb containing anti-CD3 and anti-c-*erbB*-2 were prepared. OKT-3 mAb was chemically conjugated with anti-c-*erbB*-2 mAb (SER-4) by incubation with a thiol activating reagent 5, 5′-dithio-bis-2-nitrobenzoic acid (DTNB, Sigma, St. Louis MO) as described previously (Nitta *et al.*, 1990*a*; Nitta *et al.*, 1990*b*; Nishimura *et al.*, 1991).

*Cytotoxicity assay*

The cytotoxicity of the expanded CD4$^+$ T cells was determined by 4 h–$^{51}$Cr release assay as described previously (Nishimura *et al.*, 1987). Briefly, $2.5 \times 10^3$ $^{51}$Cr-labeled target cells were incubated with $5 \times 10^4$ effector cells in the presence or absence of OKT-3 mAb (5 $\mu$g ml$^{-1}$) or BsAb (0.5 $\mu$g ml$^{-1}$) for 4 h. After 4 h, 100 $\mu$l of culture supernatants were removed to determine the cytotoxicity of effector cells. Percent cytotoxicity was determined by the following formula; % cytotoxicity = $^{51}$Cr release with effector cells − spontaneous $^{51}$Cr release/$^{51}$Cr release with 0.1 N HCl − spontaneous $^{51}$Cr release. OKT-3, OKT-4 and OKT-8 hybridoma cell, Fc-receptor (FcR) positive Daudi (Barkitt lymphoma), K562 (preerythroblastic cell), U937 (monocyte-related cell) and FcR negative IMR-32 glioma cells were used as target cells. NIH-3T3, SV11 (NIH-3T3 transfected with normal c-*erbB*-2 gene), A4–15 (NIH-3T3 cells transfected with mutated c-*erbB*-2 gene), RAScD (NIH-3T3 cells transfected with EJ *ras* gene) and KATOIII (human gastric cancer) were used for the targeting of CD4$^+$ helper/killer T cells. The oncogene transfectants were kindly donated by Dr T. Yamamoto (Tokyo University School of Medicine, Tokyo, Japan) (Masuko *et al.*, 1989).

### Results

### Proliferation of freshly isolated human CD4+ T cells by immobilised OKT-3 mAb plus rIL-2

Human $CD4^+$ T cells which were freshly isolated from PBMC of breast cancer patient using FACStar or Dynabeads were plated into 96 well plates and their proliferative responses to IL-2, immobilised OKT-3 mAb or IL-2 plus immobilised OKT-3 mAb were determined. As shown in Figure 2, FACStar-sorted $CD4^+$ T cells did not respond to IL-2 or immobilised OKT-3 mAb alone. However, they showed a marked proliferative response in the presence of IL-2 plus immobilised OKT-3 mAb. In contrast to FACStar-sorted $CD4^+$ T cells, $CD4^+$ T cells separated with Dynabeads showed a marked responsiveness to immobilised OKT-3 mAb alone. The OKT-3 mAb-induced proliferation of Dynabeads-separated $CD4^+$ T cells was further enhanced by adding rIL-2 into the culture. It was also notable that Dynabeads-separated $CD4^+$ T cells revealed higher level of proliferative responses to immobilised OKT-3 mAb plus rIL-2 than $CD4^+$ T cells isolated by FACStar.

### Large-scale expansion of CD4+ T cells

$CD4^+$ T cells were isolated from 20 ml of blood of two breast and two colon cancer patients by Dynabeads. Then, the separated $CD4^+$ T cells ($1-3 \times 10^6$) were cultured with immobilised OKT-3 mAb plus rIL-2. After 6–7 day-culture, the activated $CD4^+$ T cells ($2 \times 10^8$) were restimulated with immobilised OKT-3 mAb and inoculated into CRTC bags. As illustrated in Figure 3, $CD4^+$ T cells showed rapid growth in CRTC bag in the presence of rIL-2, and their numbers increased to over $3-5 \times 10^9$ within 14–16 days. This large-scale culture yielded an approximate 3000-fold increase in cell numbers after 16 day-culture.

### Helper and killer functions of the expanded CD4+ T cells

The functions of the expanded $CD4^+$ T cells were examined after 16 days of culture. The cells produced large amounts of IL-2 after stimulation with PMA plus A23187, but not with either alone (Table I). The OKT-3 mAb-coated Dynabeads also stimulated $CD4^+$ T cells to produce IL-2. Addition of PMA into the culture enhanced OKT-3 mAb-induced IL-2 production of $CD4^+$ T cells. Thus, the $CD4^+$ T cells expanded by our culture system retained their ability to produce IL-2, which was triggered through T cell receptor-CD3 complex or intracellular signals. The IL-2 activity in the culture supernatants was completely neutralised by anti-IL-2 polyclonal antibody (data not shown).

The killer functions of the activated $CD4^+$ T cells were also determined. As shown in Figure 4, the activated $CD4^+$ T cells lysed anti-CD3 mAb-producing hybridoma cells, but not anti-CD4 or anti-CD8 mAb-producing hybridoma cells. These results indicated that $CD4^+$ T cells lysed hybridoma cells in anti-CD3 mAb-dependent manner. Therefore, we also examined the cytotoxicity of expanded $CD4^+$ T cells against various tumour cell lines in the presence or absence of OKT-3 mAb. OKT-3 mAb-induced cytotoxicity has been demonstrated to be only achieved by direct binding of the FcR on

**Figure 2** Proliferative responses of human $CD4^+$ T cells isolated with FACStar or Dynabeads. Human $CD4^+$ T cells were isolated using FACStar (open bars) or Dynabeads (hatched bars) as described in Materials and methods. The isolated $CD4^+$ T cells were cultured in 96 well plates with medium, 2000 U $ml^{-1}$ of rIL-2, immobilised OKT-3 mAb or immobilised OKT-3 mAb plus rIL-2. The proliferative responses of the cells were determined by pulsing with $^3$H-TdR for 4 h at 3 days after culture.

**Figure 3** The growth curve of $CD4^+$ helper/killer cells in a CRTC bag. $CD4^+$ T cells were isolated from colon cancer patients (No 1 and No 3) or breast cancer patients (No 2 and No 4) using Dynabeads. The isolated $CD4^+$ T cells ($1-3 \times 10^6$) were cultured with immobilised OKT-3 mAb plus IL-2. At 3 days after culture, Dynabeads were removed from the culture using Dynal magnet. When the cells were increased to $1-2 \times 10^8$ in cell number (6–7 days after culture), the cells were restimulated with immobilised OKT-3 mAb in 96 well plates for 24 h, and then inoculated into CRTC bag. The cell numbers in CRTC bag was counted until 16 days after the initiation of culture.

**Table I** Production of IL-2 by expanded $CD4^+$ helper/killer T cells

| | Patient No. | | | |
|---|---|---|---|---|
| *Stimulator* | *1* | *2* | *3* | *4* |
| None | <2 | <2 | <2 | <2 |
| PMA alone | <2 | <2 | <2 | <2 |
| A23187 alone | <2 | <2 | <2 | <2 |
| PMA + A23187 | 64.0 | 73.5 | 73.8 | 79.0 |
| OKT-3 mAb bound Dynabeads | 58.0 | 46.0 | 58.0 | 20.0 |
| OKT-3 mAb bound Dynabeads + PMA | 68.0 | 56.0 | 80.0 | 78.6 |

At the end of culture, the cells were stimulated with various stimulators and their ability to produce IL-2 was measured using IL-2-dependent HT-2 cells, as described in Materials and methods. IL-2 activity is expressed by a unit per $2 \times 10^6$ cells. The unit of IL-2 was defined by the reciprocal dilution of IL-2 sample that evoked 50% of the maximum proliferation (c.p.m.) of HT-2 cells. Recombinant IL-2 which unit was already defined by NIH standard sample was used for the determination of IL-2 unit of sample.

**Figure 4** OKT-3 mAb-induced cytotoxicity against a variety of tumour cells by expanded CD4$^+$ helper/killer T cells. CD4$^+$ helper/killer cells were induced from four different tumour patients described in the legend of Figure 3. Cytotoxicity against various tumour cells was measured by a $^{51}Cr$ release assay in the presence (hatched bars) or absence (open bars) of 5 $\mu g\ ml^{-1}$ of OKT-3 mAb. Effector to target cell ratio was 20:1. No exogenous OKT-3 mAb was added into the culture when hybridoma cells (OKT-3, OKT-4, OKT-8) were used as target cells, but each hybridoma cell could produce anti-CD3, anti-CD4 or anti-CD8 mAb, respectively, during cytotoxicity assay.

target cells to Fc posion of OKT-3 mAb (Seventer *et al.*, 1987). Judging from their sensibility to anti-CD3 mAb induced cytotoxicity, K562, Daudi and U937 cells, but not IMR-32 glioma cells, have demonstrated to express FcR on their cell surface (Seventer *et al.*, 1987 and Nitta *et al.*, 1990*b*). Inconsistent with these results, the activated CD4$^+$ T cells lysed K562. Daudi and U937 cells in the presence of OKT-3 mAb. However, IMR-32 glioma cells were resistant to OKT-3 mAb-induced cytotoxicity of CD4$^+$ T cells (Figure 4). The resistance of IMR-32 against CD4$^+$ T cells-mediated killing was not derived from their unresponsiveness to killing because IMR-32 were lysed by CD4$^+$ T cells by adding anti-CD3 × anti-glioma bispecific antibody (Nishimura *et al.*, 1992*a*). From these results, it was demonstrated that the activated CD4$^+$ T cells with immobilised OKT-3 mAb plus rIL-2 had both helper and killer functions. Such killer function of CD4$^+$ T cells were not detected in freshly isolated CD4$^+$ T cells as reported previously (Nishimura *et al.*, 1992*a*).

*In* vitro *targeting of the expanded CD4$^+$ helper/killer T cells by using anti-CD3* × *anti-c-*erbB*-2 BsAb*

We tried *in vitro* targeting of the expanded CD4$^+$ helper/killer T cells to various kinds of tumour cell lines by using anti-CD3 × anti-c-*erB*-2 BsAb. As shown in Figure 5, the expanded CD4$^+$ helper/killer T cells showed slight cytotoxicity against tumour cell lines in the absence of BsAb. However, addition of BsAb into the culture resulted in the induction of a strong cytotoxicity against c-*erbB*-2 transfectants (A4–15 and SV-11) and c-*erbB*-2 expressing tumour cell line, KATOIII. In contrast, native NIH-3T3, EJ *ras*-transfectant (RAScD) or c-*erbB*-2 negative tumour cell line, Daudi B lymphoma cells were resistant to CD4$^+$ helper/killer cells even in the presence of BsAb. Thus, these results indicated that it was possible to develop an efficient strategy for the targeting of CD4$^+$ helper/killer T cells to tumour cells using BsAb.

**Figure 5** Specific *in vitro* targeting of expanded $CD4^+$ helper/killer T cells by using anti-CD3 × anti-c-*erbB*-2 BsAb. $CD4^+$ helper/killer cells were induced from four different tumour patients described in the legend of Figure 3. The cytotoxicity was determined by 4 h-$^{51}$Cr release assay in the presence (hatched bars) or absence (open bars) of anti-c-*erbB*-2 BsAb (0.5 $\mu$g ml$^{-1}$). Effector to target cell ratio was 20:1. Oncogene transfectant (SV11, A4–15, RAScD) and human tumour cells (KATOIII, Daudi B lymphoma) were used as target cells. The cell surface expression of c-*erbB*-2 was determined by FACScan.

*Flow cytometry analysis of $CD4^+$ helper/killer T cells*

The surface markers of the expanded $CD4^+$ helper/killer T cells were analysed by flow-cytometry technique at the end of culture. As shown in Table II, the activated cells continued to express CD4 antigen. Moreover, it was demonstrated that most of the $CD4^+$ T cells expressed CDw29 and CD45RO, though a small percentage of the cells also expressed CD45RA antigen. These results indicated that stimulation of $CD4^+$ T cells with OKT-3 mAb and rIL-2 resulted in a selective expansion of helper-inducer subpopulation of $CD4^+$ T cells.

**Table II** Cell surface markers of expanded $CD4^+$ helper/killer T cells

| *Patient No.* | *CD4/CD8*[a] | *CD4, CDw29*[b] | *CD45RO/CD45RA*[c] |
|---|---|---|---|
| 1 | 98/1 | 99 | 96/1 |
| 2 | 95/2 | 97 | 89/10 |
| 3 | 97/1 | 98 | 90/6 |
| 4 | 96/1 | 99 | 98/3 |

[a]Percentage of cells expressing either CD4 or CD8 was determined by flow cytometric analysis as described in Materials and methods. [b]Percentage of cells expressing both CD4 and CDw29. [c]Percentage of cells expressing either CD45RO or CD45RA.

### Discussion

This paper presents an efficient method for the large-scale culture of human $CD4^+$ helper/killer T cells from Dynabeads-isolated $CD4^+$ T cells. As reported previously (Nakamura *et al.*, 1991), freshly isolated $CD4^+$ T cells by FACStar can not respond to rIL-2 because of their lack of p75 IL-2R expression. However, stimulation of $CD4^+$ T cells with immobilised OKT-3 mAb induced p75 IL-2R expression and their marked proliferative response to rIL-2 (Nishimura *et al.*, 1992*a*). As shown in Figure 2, FACStar sorted $CD4^+$ T cells showed no significant proliferative response to immobilised OKT-3 mAb in the absence of IL-2. In contrast, CD4$^+$ T cells isolated with Dynabeads revealed a significant proliferative response to immobilised OKT-3 mAb in the absence of rIL-2 (Figure 2). Crosslinking of cell-bound OKT-4 mAb with anti-mouse Ig coupled-Dynabeads resulted in the conversion of OKT-4 mAb from soluble to solid phase. Therefore, Dynabeads-separated CD4$^+$ T cells may be activated through both CD3 and CD4 molecules. Our results are supported by the results of Emmrich *et al.* (1987) who reported that double immobilisation of anti-CD3 and anti-CD4 induced higher proliferation of CD4$^+$ T cells compared with single immobilisation of anti-CD3 mAb. Thus, the separation procedure of CD4$^+$ T cells using Dynabeads has two advantages over a method using a cell sorter; (1) The procedure is simple and rapid; (2) Dynabeads preparation causes augmented proliferation of CD4$^+$ T cells against immobilised anti-CD3 plus IL-2.

As reported previously (Noto *et al.*, 1989), we demonstrated that large numbers of LAK cells could be expanded in CRTC culture bag which was developed by us. We have now shown that the CRTC culture bag is suitable for the expansion of CD4$^+$ T cells. It has been reported that culture of PBMC by culture plate in the presence of rIL-2 alone yielded about 10–20 fold increase in cell numbers, and culture with immobilised anti-CD3 mAb plus rIL-2 yielded a 500 fold increase in cell numbers (Anderson *et al.*, 1988). We also demonstrated that culture of isolated CD4$^+$ T cells with immobilised OKT-3 mAb plus rIL-2 caused 200 fold increase from $10^6$ to $2 \times 10^8$ cells in 6–7 days culture. As previously reported by us, CD4$^+$ T cells could be expanded for over a month in a split culture using 96 well plate. Because CD4$^+$ T cells grew in 96 well plates at the same growth rate as in the CRTC bags, it is theoretically possible to expand $10^9$ levels of CD4$^+$ T cells by general tissue culture method if we use hundreds of culture plates and huge volumes of culture medium. As shown in Figure 3, we demonstrate that $10^9$ levels of CD4$^+$ T cells can be expanded using a simple CRTC culture bag system without changing medium. Our established large-scale culture system, which consists of initial culture using 96 well plates and late culture using the CRTC bag, yielded a 3000 fold increase in cell number within 16 days of culture. Therefore, it is possible to obtain $10^{10}$ isolated CD4$^+$ T cells in less than 20-day culture. We believe that our established culture system is the simplest method for the large-scale expansion of CD4$^+$ helper/killer T cells. In this paper, we showed four representative results using CD4$^+$ T cells isolated from tumour patients. Generally, tumour-bearing hosts reveal suppressed immune responses (Burgar *et al.*, 1984), whereas the separated CD4$^+$ T cells from tumour patients showed the same level of proliferative responses to immobilised OKT-3 plus rIL-2 as CD4$^+$ T cells isolated from healthy donors (Nishimura *et al.*, 1992*a*). Actually, CD4$^+$ T cells obtained from both tumour patients and healthy donors showed the same growth curve in CRTC bags (data not shown).

As shown in Table I and Figure 4, the CD4$^+$ T cells expanded in CRTC bag also showed both IL-2 producing activity and OKT-3 mAb-mediated cytotoxicity. Triggering of the expanded CD4$^+$ T cells through CD3 molecule stimulated both helper and killer functions. Therefore, if we can target the CD4$^+$ helper/killer cells to tumour cells using anti-CD3 × anti-tumour bispecific antibody, the targeted CD4$^+$ T cells act as both killer and IL-2 producer at tumour local site. As shown in Figure 5, CD4$^+$ helper/killer cells expanded by our established methods were specifically targeted to c-*erbB*-2 positive tumour cell lines *in vitro* using anti-CD3 × anti-c-*erbB*-2 BsAb. We have an evidence that CD4$^+$ helper/killer cells showed a strong therapeutic efficacy against human colon cancer cells implanted into nude mice by combination with BsAb (Nishimura *et al.*, 1992*b*). Recent results demonstrated that CD4$^+$ tumour infiltrating lymphocytes were specifically targeted to tumour site compared with LAK cells (Grimm *et al.*, 1991). Several animal experiments also demonstrated that adoptive tumour immunotherapy using CD4$^+$ T cells was effective (Bookman *et al.*, 1987). The therapeutic advantage of CD4$^+$ helper/killer cells is that they may function both as effector cells and helper cells at local site of tumour. We believe that our established simple method for the large-scale culture of CD4$^+$ helper/killer will be available for new trials of adoptive immunotherapy (helper/killer therapy).

This work was supported in part by the Grant-in-Aid for Cancer research from the Ministry of Education, Science and Culture, Japan and Tokai University Medical School Research Aid, Kanagawa Academy Science and Technology Foundation, and Japanese Foundation for Multidisplinary treatment of Cancer, and a Grant-in-Aid for a DNA project from Tokai University School of Medicine.

### References

ANDERSON, P.M., BACH, F.H. & OCHOA, A.C. (1988). Augmentation of cell number and LAK activity in peripheral blood mononuclear cells activated with anti-CD3 and interleukin-2. Preliminary results in with acute lymphocytic leukemia and neuroblastoma. *Cancer Immunol. Immunother.*, **27**, 82.

BOOKMAN, M.A., SWERDLOW, R. & MATIS, A. (1987). Adoptive chemoimmunotherapy of murine leukemia with helper T lymphocytes. *J. Immunol.*, **139**, 3166.

BUBENIK, J., SIMOVA, J. & JANDLOVA, T. (1990). Immunotherapy of cancer using local administration of lymphoid cells transformed by IL-2 cDNA and constitutively producing IL-2. *Immunol. Lett.*, **23**, 287.

BULLBERG, M., POBOR, G., BANDEIRA, A., LARSSON, E.-L. & COUTINBO, A. (1983). Differential requirements for activation and growth of unprimed cytotoxic and helper T lymphocytes. *Eur. J. Immunol.*, **13**, 719.

BURGAR, C.J., ELGERT, K.D. & FARRAR, W.L. (1984). Interleukin 2 (IL-2) activity during tumor growth: IL-2 production kinetics, absorption of and response exogenous IL-2. *Cell Immunol.*, **84**, 228.

CHEEVER, M.A., GREENBERG, P.D., FEFER, A. & GILLIS, S. (1982). Augmentation of the anti-tumor therapeutic efficacy of long-term cultured T lymphocytes by *in vivo* administration of purified interleukin 2. *J. Exp. Med.*, **155**, 968.

CHIBA, K., NISHIMURA, T. & HASHIMOTO, Y. (1985). Stimulated rat T cell-derived inhibitory factor for cellular DNA synthesis (STIF) I. Isolation and characterization. *J. Immunol.*, **134**, 1019.

EMMRICH, F., KANZ, L. & EICHMANN, K. (1987). Cross-linking of the T cell receptor complex with the subset-specific differentiation antigen stimulates interleukin 2 receptor expression in human CD4 and CD8 T cells. *Eur. J. Immunol.*, **17**, 529.

FEARON, E.R., PARDOLL, D.M., ITAYA, T., GOLUMBEK, P., LEVITSKY, H.I., SIMONS, J.W., KARASUYAMA, H., VOGELSTEIN, B. & FROST, P. (1990). Interleukin-2 production by tumor cells bypasses T helper function in the generation of an antitumor response. *Cell*, **60**, 397.

GAUDERNACK, G., LEIVESTAD, T., UGELSTAD, J. & THORSBY, E. (1986). Isolation of pure functionally active CD8$^+$ T cells. Positive selection with monoclonal antibodies directly conjugated to monosized magnetic microspheres. *J. Immunol. Methods*, **90**, 179.

GRIMM, E.A., BRUNER, J.M., CARINHAS, J., KÖPPEN, J.A., LOUDON, W.G., OWEN-SCHAUB, L., STECK, P.A. & MOSER, R.P. (1991). Characterization of interleukin-2-initiated versus OKT3-initiated human tumor-infiltrating lymphocytes from glioblastoma multiforme: growth characteristics, cytolytic activity, and cell phenotype. *Cancer Immunol. Immunother.*, **32**, 391.

MAEDA, M., NOMA, T., HAMA, K. & HONJYO, T. (1988). Application of a human T cell line derived from a Sezary syndrome patient for human interleukin 4 assay. *Immunol. Lett.*, **18**, 247.

MASUKO, T., SUGAHARA, K., KOZONO, M., OTSUKI, S., AKIYAMA, T., YAMAMOTO, T., TOYOSHIMA, K. & HASHIMOTO, Y. (1989). A murine monoclonal antibody that recognizes an extracellular domain of the human c-erbB-2 protooncogene product. *Jpn. J. Cancer*, **80**, 10.

MULE, J.J., SHU, S., SCHWARZ, S.L. & ROSENBERG, S.A. (1984). Adoptive immunotherapy of established pulmonary metastases with LAK cells and recombinant interleukin-2. *Science*, **225**, 1487.

NAKAMURA, Y., WATANABE, K., NOTO, T., TAJIMA, T. & YAMAMURA, M. (1989). A new compact and cell dense continous culture system. *J. Immunol. Methods*, **118**, 31.

NAKAMURA, Y., NISHIMURA, T., TOKUDA, Y., KOBAYASHI, N., WATANABE, K., NOTO, T., MITOMI, T., SUGAMURA, K. & HABU, S. (1991). Macrophage-T cell interaction is essential for the induction of p75 interleukin 2 (IL-2) receptor and IL-2 responsiveness in human $CD4^+$ T cells. *Jpn. J. Cancer Res.*, **82**, 257.

NISHIMURA, T., TOGASHI, Y., GOTO, M., YAGI, H., UCHIYAMA, Y. & HASHIMOTO, Y. (1986). Augmentation of the therapeutic efficacy of adoptive tumor immunotherapy by *in vivo* administration of slowly released recombinant interleukin 2. *Cancer Immunol. Immunother.*, **21**, 12.

NISHIMURA, T., BURAKOFF, S.J. & HERRMANN, S.H. (1987). Protein kinase C required for cytotoxic T lymphocyte triggering. *J. Immunol.*, **130**, 2888.

NISHIMURA, T., UCHIYAMA, Y. & HASHIMOTO, Y. (1988). *In vivo* generation of lymphokine-activated killer cells by sensitization with interleukin 2-producing syngeneic T lymphoma cells. *Cell Immunol.*, **112**, 220.

NISHIMURA, T., TAKEUCHI, Y., ICHIMURA, Y., GAO, X., AKATUKA, A., TAMAOKI, N., YAGITA, H., OKUMURA, K. & HABU, S. (1990). Thymic stromal cell clone with nursing activity supports the growth and differentiation of murine CD4 + 8 + thymocytes *in vitro*. *J. Immunol.*, **134**, 4012.

NISHIMURA, T., NAKAMURA, Y., TAKEUCHI, Y., GAO, X., TOKUDA, Y., OKUMURA, K. & HABU, S. (1991). Bispecific antibody-directed antitumor activity of human $CD4^+$ helper/killer T cells induced by anti-CD3 monoclonal antibody plus interleukin 2. *Jpn. J. Cancer Res.*, **82**, 1207.

NISHIMURA, T., NAKAMURA, Y., TAKEUCHI, Y., TOKUDA, Y., IWASAWA, M., KAWASAKI, A., OKUMURA, K. & HABU, S. (1992*a*). Generation, propagation and targeting of human $CD4^+$ helper/killer T cells induced by anti-CD3 monoclonal antibody plus recombinant interleukin 2: An efficient strategy for adoptive tumor immunotherapy. *J. Immunol.*, **148**, 285.

NISHIMURA, T., NAKAMURA, Y., TSUKAMOTO, H., TAKEUCHI, Y., TOKUDA, Y., IWASAWA, M., YAMAMOTO, T., MASUKO, T., HASHIMOTO, Y. & HABU, S. (1992*b*). Human c-erbB-2 protooncogene products as a target for bispecific antibody-directed adoptive tumor immunotherapy. *Int. J. Cancer*, **51**, 1.

NITTA, T., SATO, K., YAGITA, H., OKUMURA, K. & ISHII, S. (1990*a*). Preliminary trial of specific targeting therapy against malignant glioma. *Lancet*, **335**, 368.

NITTA, T., SATO, K., OKUMURA, K. & ISHII, S. (1990*b*). Induction of cytotoxicity in human T cells coated with anti-glioma × anti-CD3 bispecific antibody against human glioma cells. *J. Neurosurg.*, **72**, 476.

NOTO, T., TOKUDA, Y., NAKAMURA, Y., SUZUKI, A., WATANABE, K., YAMAMURA, M., TAJIMA, T., MITOMI, T. & NISHIJIMA, K. (1989). A new high-yield continuous cell-culture system for lymphokine activated killer cells. *Cancer Immunol. Immunother.*, **30**, 1.

OHASHI, Y., TAKESHITA, T., NAGATA, K., MORI, S. & SUGAMURA, K. (1989). Differential expression of the IL-2 receptor subunits, p55 and p75 on various populations of primary peripheral blood mononuclear cells. *J. Immunol.*, **143**, 3548.

ROSENBERG, S.A., LOTZE, M.T., MUUL, L.M., LEITMAN, S., CHANG, A.E., ETTINGHAUSEN, S.E., MATORY, Y.L., SKIBBER, J.M., SHILONI, E., VETTO, J.T., SEIPP, C.A., SIMPSON, C. & REICHERT, C.M. (1985). Observations on the systemic administration of autologous lymphokine-activated killer cells and recombinant interleukin-2 to patients with metastatic cancer. *N. Eng. J. Med.*, **313**, 1485.

SEVENTER, G.A., KUIJPERS, K.C., VAN LIER, R.A.W., DE GROOT, E.R., AARDEN, L.A. & MELIEF, C.J.M. (1987). Mechanism of inhibition and induction of cytolytic activity in cytotoxic T lymphocytes by CD3 monoclonal antibody. *J. Immunol.*, **139**, 2545.

TAYLOR, D.S., REED, J.C. & NOWELL, P.C. (1985). Stimulation and inhibition of human T cell subsets by wheat germ agglutinin. *J. Immunol.*, **134**, 3756.